# Hyperlipidemia Is Associated with Chronic Urticaria: A Population-Based Study

**DOI:** 10.1371/journal.pone.0150304

**Published:** 2016-03-10

**Authors:** Shiu-Dong Chung, Kuo-Hsien Wang, Ming-Chieh Tsai, Herng-Ching Lin, Chao-Hung Chen

**Affiliations:** 1 Department of Surgery, Far Eastern Memorial Hospital, Banciao, Taipei, Taiwan; 2 Graduate Program in Biomedical Informatics, College of Informatics, Yuan-Ze University, Chung-Li, Taiwan; 3 Sleep Research Center, Taipei Medical University Hospital, Taipei, Taiwan; 4 Department of Dermatology, Taipei Medical University and Hospital, Taipei, Taiwan; 5 School of Healthcare Administration, Taipei Medical University, Taipei, Taiwan; 6 Department of Internal Medicine, Cathay General Hospital, Hsinchu Branch, Hsinchu, Taiwan; 7 Department & Institute of Physiology, National Yang-Ming University, Taipei, Taiwan; 8 Department of Cosmetic Applications and Management, Mackay Junior College of Medicine, Nursing, and Management, Taipei, Taiwan; 9 Department of Thoracic Surgery, MacKay Memorial Hospital, Taipei, Taiwan; Osaka University Graduate School of Medicine, JAPAN

## Abstract

The etiology of chronic urticaria (CU) is diverse, with chronic infections and inflammation being reported as considerable contributing factors. Although the prevalence of metabolic syndrome was found to be significantly elevated in patients with CU, no one has specifically estimated the effects on CU following hyperlipidemia. This study aimed to examine the association between hyperlipidemia and CU using a population-based dataset in Taiwan. This study included 9798 adults with CU as cases and 9798 sex- and age-matched controls. These patients were examined for whether they had received a prior diagnosis of hyperlipidemia. We used conditional logistic regression analyses to calculate the odds ratio (OR) and its corresponding 95% confidence interval (CI) for having been previously diagnosed with hyperlipidemia between cases and controls. In total, 7066 (36.1%) patients had received a prior diagnosis of hyperlipidemia, including 4287 (43.8%) among CU cases and 2779 (28.4%) among controls. The conditional logistic regression revealed that the OR of prior hyperlipidemia for cases was 1.97 (95% CI: 1.85~2.09) compared to the controls. Furthermore, compared to patients without CU, patients with CU independently experienced a 1.65-fold (95% CI = 1.55~1.76; *p*<0.001) increased risk of having a prior hyperlipidemia diagnosis, after adjustments were made. We concluded that CU was associated with having received a prior diagnosis of hyperlipidemia.

## Introduction

Chronic urticaria (CU) is a distressing and common skin disorder characterized by the persistent or recurrent appearance of wheals and/or angioedema lasting for more than 6 weeks [1]. The lifetime prevalence of CU was estimated to be 1.8% for the adult population, with a period prevalence (in the past 12 months) of 0.6%~0.8% [2–4]. Although the natural course of CU is self-limited, with spontaneous remissions and intermittent relapses, impairment to the quality of life is usually severe [5,6].

More than 80% of adults and children with CU are unable to attribute its occurrence to external allergic causes or disease processes [7–9]. Several theories regarding the pathogenesis of idiopathic CU were proposed (e.g., stress, allergies to food, and autoantibody production against the immunoglobulin E (IgE) receptor) [10]. Nevertheless, the etiology of CU is very diverse with several possible triggers having been identified. In the 2010 Asian Academy of Dermatology and Venereology Study Group (AADV) consensus guidelines [11], chronic infection and inflammation were reported to be significant contributing factors to CU. Chronic inflammatory processes, especially gastritis and inflammation of the bile duct or gall bladder, were proposed as being involved in the induction of CU [1].

Indeed, CU is considered to be a chronic inflammatory disease as depicted by cutaneous mast cell degranulation, and infiltrating T cells, eosinophils, and neutrophils. Increased circulating levels of C-reactive protein (CRP) and proinflammatory cytokines, such as interleukin (IL)-6, tumor necrosis factor (TNF)-α, and matrix metalloproteinase (MMP)-9 were observed among patients with CU [12–14].

Metabolic syndrome is a combination of central obesity, dyslipidemia, elevated blood pressure, and elevated fasting plasma glucose. Among these, hyperlipidemia was implicated as being involved in inflammatory processes associated with increased risks of subsequent atherosclerosis and cardiovascular diseases (CVDs), major causes of death in both developed and developing countries.

A systemic pro-inflammatory and pro-coagulating state, illustrated by elevated levels of inflammatory markers such as IL-1, IL-6, TNF, and CRP, was reported among individuals with metabolic syndrome [15]. Previous studies identified systemic inflammation and activated coagulation signaling in patients with CU and metabolic syndrome [13,16–19]. Moreover, the prevalence of metabolic syndrome was significantly greater in patients with CU than in healthy controls [20]. In the examination of serum lipids and fatty acids among 12 normal participants and 23 patients with CU, a relationship between CU and serum lipids and fatty acids was reported [21]. The omega 6 (n-6) and omega 3 (n-3) series of polyunsaturated fatty acids and lipid peroxidation were implicit to be one of the mediators in CU.

Nevertheless, no study has yet specifically determined an association between hyperlipidemia and CU. The objective of this population-based study was thus to examine whether CU was associated with having received a prior diagnosis of hyperlipidemia.

## Methods

### Database

This case-control study retrieved data from the Longitudinal Health Insurance Database 2005 (LHID2005). The LHID2005 comprises medical claims and registration files for 1,000,000 enrollees under the Taiwan National Health Insurance (NHI) program. The Taiwan National Health Institute (TNHI) randomly selected these 1,000,000 enrollees from all enrollees listed in the 2005 Registry of Beneficiaries (*n* = 25.68 million). Everyone who was a beneficiary of the NHI program during any period in 2005 is in the population for random sampling. Prior studies and the TNHI have reported the high validity of data from the NHI program.

### Selection of cases and controls

We first selected all patients who had received a first-time diagnosis of CU (ICD-9-CM code 708.8) in an ambulatory care visit (including outpatient departments of hospitals and clinics) between January 1, 2002 and December 31, 2012 (*n* = 24,348). Since the administrative datasets are always criticized for their poor diagnosis validity, this study only included 11,101 patients who had at least two principal diagnoses of CU coded in their medical claims, with at least one of those diagnoses being made by a certified dermatologist in order to increase the validity of the CU diagnoses. We then excluded 1303 patients aged younger than 18 years in order to limit the study sample to the adult population. Ultimately, 9798 patients with CU were included as cases. We assigned the first ambulatory care visit for receiving a diagnosis of CU as the index date.

We likewise retrieved controls from the remaining enrollees of the LHID2005. We first excluded all enrollees who had a history of CU since inauguration of the NHI program in 1995. We then randomly selected 9798 controls (one control per case) to match the cases by sex, age group (18~29, 30~39, 40~49, 50~59, 60~69, and ≥70 years), and index year. In this study, the year of the index date for cases was the year in which the cases received their first diagnosis of CU. For controls, the index year was simply a matched year in which the controls had a medical utilization. We assigned the first medical utilization in the index year as the index date for controls.

### Exposure assessment

All hyperlipidemia cases were identified based on ICD-9-CM codes 272.0, 272.1, 272.2, 272.3, and 272.4. In this study, we only considered those hyperlipidemia cases that had received more than two hyperlipidemia diagnoses prior to the index date. This was done in order to increase the validity of the hyperlipidemia diagnoses sourced from the administrative database.

### Statistical analysis

The SAS statistical package (SAS System for Windows, Version 8.2, Cary, NC) was used to perform all analyses in the dataset of this study (S1 Data). We used Chi-squared tests to compare differences in sociodemographic characteristics, including monthly income, geographic region, and urbanization level, between cases and controls. We also used conditional logistic regression analyses (conditioned on sex, age, and the index year) to calculate the odds ratio (OR) and its corresponding 95% confidence interval (CI) for having been previously diagnosed with hyperlipidemia between cases and controls. In addition to this, we took potential risk factors for CU including atopic disorders (asthma, ICD-9-CM code 493; allergic rhinitis, ICD-9-CM code 477; and atopic dermatitis, ICD-9-CM codes 69)), autoimmune diseases (rheumatoid arthritis, ICD-9-CM code 714.0) and ankylosing spondylitis (ICD-9-CM codes 720 or 720.0), systemic lupus erythematosus (ICD-9-CM code 710.0)), and pneumonia within 1 year prior to the index date into consideration in the regression models. The conventional *p*≤0.05 was used to assess statistical significance.

## Results

Of the sampled patients, the mean age was 43.9 years with a standard deviation of 16.5 years, and 40.1% were males. After matching for sex, age, and index date, it can be seen from Table 1 that there was a significant difference in geographic region, monthly income, urbanization level, atopic disorders, and autoimmune diseases between cases and controls (all *p*<0.001). No significant difference in the prevalence of pneumonia was observed between cases and controls (*p* = 0.539).

**Table 1:** Demographic characteristics of patients with chronic urticaria (CU) and controls in Taiwan (n = 19,596).

Variable	Patients with CU (*n* = 9798)		Controls (*n* = 9798)		*p* value
	Total No.	%	Total No.	%	
Male	3929	40.1	3929	40.1	>0.999
Age, mean (SD), years	43.9 (16.5)		44.0 (16.1)		0.940
Number of Outpatient visits, mean (SD)					
1 year before index date	24.9 (20.8)		17.2 (16.6)		<0.001
2 years before index date	48.5 (40.0)		33.7 (31.1)		<0.001
3 years before index date	70.9 (57.7)		49.4 (45.5)		<0.001
Urbanization level					<0.001
1 (most urbanized)	3159	32.3	3166	32.3	
2	2796	28.5	2919	29.8	
3	1555	15.9	1630	16.6	
4	1238	12.6	1231	12.6	
5 (least urbanized)	1050	10.7	852	8.7	
Monthly income					<0.001
≤ NT$15,840	4172	42.6	3712	37.9	
NT$15,841~25,000	3154	32.2	3260	33.3	
≥ NT$25,001	2472	25.2	2826	28.8	
Geographic region					<0.001
Northern	4795	48.9	4346	44.4	
Central	2197	2197	2457	25.3	
Southern	2607	2607	2725	27.8	
Eastern	199	199	252	2.6	
Atopic disorders	3312	33.8	2338	23.9	<0.001
Autoimmune diseases	336	3.4	219	2.2	<0.001
Pneumonia	17	0.2	15	0.2	0.724

The average exchange rate in 2012 was US$1.00≈New Taiwan (NT)$30.

Table 2 shows the prevalence of prior hyperlipidemia between cases and controls. Of the total 19,596 sampled patients, 7066 (36.1%) had received a hyperlipidemia diagnosis prior to the index date. A Chi-squared test revealed that there was a significant difference in the prevalence of prior hyperlipidemia between cases and controls (43.8% vs. 28.4%, *p*<0.001).

**Table 2:** Prevalence and odds ratios (ORs) for prior hyperlipidemia among sampled subjects.

Presence of prior hyperlipidemia	Total *(n = 19*,*596*)		Patients with chronic urticaria (*n = 9798*)		Controls (*n = 9798*)	
	*n*, %		*n*, %		*n*, %	
Yes	7066	36.1	4287	43.8	2779	28.4
Crude OR (95% CI)	—		1.97* (1.85~2.09)		1.00	
Adjusted OR (95% CI) ^a^	—		1.65* (1.55–1.76)		1.00	

Notes: CI, confidence interval; the OR was calculated by a conditional logistic regression which was conditioned on gender, age, and index year;

* *p*<0.001.

^a^ Adjustments were made for patient’s urbanization level, monthly income, geographic region, atopic disorders, autoimmune diseases and the number of outpatient visits within one year before index date.

The ORs and their corresponding 95% CIs for having been previously diagnosed with hyperlipidemia between cases and controls are also presented in Table 2. The conditional logistic regression (conditioned on sex, age, and the index year) revealed that the OR of prior hyperlipidemia for cases was 1.97 (95% CI: 1.85~2.09, *p*<0.001) compared to the controls. After adjusting for geographic region, monthly income, urbanization level, atopic disorders, autoimmune diseases and the number of outpatient visits within one year before index date., the OR of having previously received a hyperlipidemia diagnosis for cases was 1.65 (95% CI: 1.55~1.76; *p*<0.001) compared to controls.

Table 3 presents the sensitivity analysis of the relationship between CU and prior hyperlipidemia. The results consistently suggested that CU was significantly associated with a prior hyperlipidemia diagnosis. We found that the respective adjusted ORs for a prior hyperlipidemia diagnosis were 1.65 (95% CI: 1.55~1.76, *p*<0.001), 1.69 (95% CI: 1.58~1.80, *p*<0.001), and 1.71 (95% CI: 1.60~1.83, *p*<0.001) for cases compared to controls, after excluding CU patients who had respectively received a hyperlipidemia diagnosis within, 1, 2, and 3 years prior to the index date.

**Table 3:** Sensitivity analysis.

Presence of prior hyperlipidemia						
	Excluding CU patients who received a hyperlipidemia diagnosis within 1 year prior to the index date *n*, %	Controls *n*, %	Excluding CU patients who received a hyperlipidemia diagnosis within 2 year prior to the index date *n*, %	Controls *n*, %	Excluding CU patients who received a hyperlipidemia diagnosis within 3 year prior to the index date *n*, %	Controls *n*, %
Yes	4126 (42.8)	2663 (27.5)	4001 (42.1)	2535 (26.5)	3898 (41.4)	2440 (25.8)
OR (95% CI)	1.97* (1.86~2.01)	1.00	2.01* (1.89~2.14)	1.00	2.04* (1.91~2.16)	1.00
Adjusted ^a^ OR (95% CI)	1.65* (1.55~1.76)	1.00	1.69* (1.58~1.80)	1.00	1.71* (1.60~1.83)	1.00

CU, chronic urticaria; CI, confidence interval; OR, odds ratio; the OR was calculated by a conditional logistic regression which was conditioned on gender, age, and year of the index date;

* *p*<0.001;

^a^ Adjustments were made for patient’s urbanization level, monthly income, geographic region, atopic disorders, autoimmune diseases and the number of outpatient visits within one year before index date.

In order to examine whether hyperlipidemia was associated with CU specifically or with dermatological diseases in general, we further identified a negative control group for investigation. We selected 7388 patients who had received a first-time diagnosis of atopic dermatitis (ICD-9-CM code 691.8) in an ambulatory care visit (including outpatient departments of hospitals and clinics) between January 1, 2013 and December 31, 2013. We then randomly selected 7388 controls (one control per case) to match the cases by sex, age group (18~29, 30~39, 40~49, 50~59, 60~69, and ≥70 years), and index year. We found that of the total 14,776 sampled patients, 9.2% had received a hyperlipidemia diagnosis prior to the index date. A Chi-squared test revealed that there was not a significant difference in the prevalence of prior hyperlipidemia between cases and controls (9.6% vs. 8.8%, p = 0.073). Atopic dermatitis, not like previous CU, was not associated with prior hyperlipidemia (OR = 1.11, 95% CI = 0.99–1.24).

## Discussion

This is the first epidemiological study in the medical literature to assess the association between hyperlipidemia and CU. We found that 7066 (36.1%) subjects had received a prior diagnosis of hyperlipidemia, including 4287 (43.8%) among CU cases and 2779 (28.4%) among the controls. Compared to patients without CU, patients with CU experienced a 1.65-fold increased risk of having a prior hyperlipidemia diagnosis, after adjusting for patient’s gender, age, index year, urbanization level, monthly income, geographic region, atopic disorders, autoimmune diseases, and the number of outpatient visits within one year before index date. By considering the time required for inflammatory processes to affect further diseases, risks of prior hyperlipidemia among CU patients were then examined after excluding those who had received a hyperlipidemia diagnosis within 1, 2, and 3 years prior to the index date. In addition, a negative control group of atopic dermatitis was used for examination. These results still suggested that CU specifically, but not dermatological diseases in general, was significantly associated with a prior hyperlipidemia diagnosis.

Previous studies proposed that chronic inflammatory processes, such as gastritis and inflammation of the bile duct or bile gland, were possible factors causing urticaria.^1^ Hyperlipidemia, with high levels of lipids (fats) in the blood including cholesterol and triglycerides, was implicated as being involved in the inflammatory processes associated with higher risks of developing atherosclerosis [22]. Our study was able to show that in addition to this, there is also an increased risk of prior hyperlipidemia among patients with CU. Although no one had previously reported hyperlipidemia being related to CU, it has been shown that there is an increased level of inflammatory markers such as IL-1, Il-6, and TNF-α in patients with CU and those with metabolic syndrome [12–15]. Additionally, a hospital-based cross-sectional study of 131 patients was performed in Korea to examine the prevalence and clinical effects of metabolic syndrome among patients with CU [20]. They found in a logistic regression analysis that the presence of metabolic syndrome, together with a high urticaria activity score, was an independent predictor of uncontrolled CU.

How hyperlipidemia is linked with CU is unclear; yet preliminary evidence suggests several possibilities. In experimental and clinical studies, inflammatory markers such as CRP, TNF-α, IL-6, nuclear factor-κβ, adhesion molecules, serum amyloid-α, and lipoprotein-associated phospholipase A2 were associated with lipids level. Most of these markers were also found among patients with CU [12–15, 23]. Furthermore, CU is generally deemed a non-allergic skin illness, induced by activation of mast cells [24], which were considered to be substantially linked with hyperlipidemia or atherosclerotic disease. Explicitly, mast cells, encompassing part of the innate and adaptive immune system [25], could have a role in inflammation of the endothelium. The role was found to be proportional to the magnitude of atherosclerotic disease [26]. Prodigious evidence was reported for the underlying importance of mast cells in atherosclerosis which accumulate in atherosclerotic lesions [27]. Pro-inflammatory stimuli, including complement protein factors C3a and C5a, cytokines, MCP-1, and oxidized low-density lipoprotein (LDL), may function to activate mast cells [28,29]. Activation of mast cells might consequently affect the occurrence of CU [24]. In general, the potential interplay between the progression of hyperlipidemia and CU may foster systemic inflammation which impacts these diseases. Our preliminary findings could not elucidate whether the elevated systemic inflammation or activation of mast cells was an epiphenomenon or played a role in the pathogenesis of CU following hyperlipidemia. More studies are needed to clarify this point.

Based upon our findings, we highlight the important point that patients with CU should be carefully evaluated for the presence of hyperlipidemia. Indeed, due to its asymptomatic features, hyperlipidemia is usually under-represented in clinical settings, and appropriate management is thus not delivered. Severe consequences including increased risks of developing atherosclerosis, heart attack, stroke, and a shorter lifespan were observed for patients with hyperlipidemia, especially those with uncontrolled disease [22]. In our study, a higher risk of prior hyperlipidemia among patients with CU was identified. Because hyperlipidemia is a major, modifiable risk factor for atherosclerosis and CVDs, we thus stress the importance of a serum examination for hyperlipidemia among the higher-risk group of patients with CU. With prompt detection and appropriate management, subsequent cardiovascular risks can be reduced. CU outcomes and patients’ quality of life might also be improved.

A particular strength of this study is the use of a nationwide population-based dataset and, therefore, this study should have been free from the effects of selection bias. Although our study leads the way in examining the association between hyperlipidemia and CU, three limitations merit attention. First, the identification of CU was based solely on disease diagnostic codes. Certain laboratory tests, such as the autologous serum skin test, whole-blood count, autoantibody detection, or IgE-test, were unavailable in our claims dataset. Second, the LHID2005 database contained patients who had sought treatment for hyperlipidemia and CU. However it must be noted that hyperlipidemia is usually asymptomatic. As such under-utilization of physical healthcare by both patients with and those without CU is possible. Although the distressing symptoms of CU would usually prompt patients to seek medical care, processes of diagnosing and treating CU in dermatology departments might not involve a serum examination for hyperlipidemia detection. This relatively non-differential misclassification of a dichotomous exposure of hyperlipidemia would possibly bias our results toward the null. Third, because we utilized a claims database, our study was unable to examine patients’ history of possible eliciting factors, such as dietary habits, the domestic environment, smoking behaviors, stress, or family history regarding urticaria and atopy, which could compromise our findings.

In conclusion, this is the first epidemiological study to identify an increased risk of a prior diagnosis of hyperlipidemia among patients with CU (S1 Table). CU greatly affects one's quality of life. Shedding light on this link might boost the earlier detection of hyperlipidemia among CU patients and promote better interventions for this disease. More studies are needed to replicate these results to elucidate the association between hyperlipidemia and CU before prevention, early identification, and better clinical outcomes can be made possible.

## Supporting Information

S1 Data Minimal dataset for this study. (XLS)

S1 Table RECORD checklist. (PDF)
